# Global impression of perceived difficulties in children and adolescents with attention-deficit/hyperactivity disorder: Reliability and validity of a new instrument assessing perceived difficulties from a patient, parent and physician perspective over the day

**DOI:** 10.1186/1753-2000-2-10

**Published:** 2008-05-28

**Authors:** Peter M Wehmeier, Alexander Schacht, Ralf W Dittmann, Manfred Döpfner

**Affiliations:** 1 Lilly Deutschland, Medical Department, Bad Homburg, Germany; 2 Department of Child and Adolescent Psychosomatic Medicine, University of Hamburg, Germany; 3 Department of Child and Adolescent Psychiatry, University of Cologne, Germany

## Abstract

**Background:**

The objective of this analysis was to evaluate the psychometric properties of a brief scale developed to assess the degree of difficulties in children with Attention-Deficit/Hyperactivity Disorder (ADHD). The Global Impression of Perceived Difficulties (GIPD) scale reflects overall impairment, psychosocial functioning and Quality of Life (QoL) as rated by patient, parents and physician at various times of the day.

**Methods:**

In two open-label studies, ADHD-patients aged 6–17 years were treated with atomoxetine (target-dose 0.5–1.2 mg/kg/day). ADHD-related difficulties were assessed up to week 24 using the GIPD. Data from both studies were combined to validate the scale.

**Results:**

Overall, 421 patients received atomoxetine. GIPD scores improved over time. All three GIPD-versions (patient, parent, physician) were internally consistent; all items showed at least moderate item-total correlation. The scale showed good test-retest reliability over a two-week period from all three perspectives. Good convergent and discriminant validity was shown.

**Conclusion:**

GIPD is an internally consistent, reliable and valid measure to assess difficulties in children with ADHD at various times of the day and can be used as indicator for psychosocial impairment and QoL. The scale is sensitive to treatment-related change.

## Background

Attention-deficit/hyperactivity disorder (ADHD) is a disorder characterized by inattention, impulsivity and hyperactivity that affects 3–7% of school-age children [1]. ADHD is associated with significant impairment of cognitive and psychosocial functioning [2,3] and quality of life (QoL) in patients and their families [4-9]. Psychostimulants and behavior therapy are known to be effective in the treatment of ADHD, as reported in the MTA study [10] and other studies (e.g. Döpfner et al. 2004) [11]. Atomoxetine is a non-stimulant treatment option for ADHD [12,13] for which efficacy and tolerability in children and adolescents has been demonstrated in a number of randomized, placebo-controlled trials [14-17], supported by a recent meta-analysis [18]. In addition, several studies have shown improvement of health-related QoL in children and adolescents treated with atomoxetine [16,19-25]. In most of these studies, investigator-rated questionnaires such as the ADHD-Rating Scale (ADHD-RS) [26,27], the Clinical Global Impression (CGI) [28,29], or the parent-rated ADHD-symptom checklists and other questionnaires such as the Child Health Questionnaire (CHQ) [30] were used. However, when assessing QoL in children and adolescents with ADHD, both symptom severity and ADHD-related difficulties may be perceived and rated differently by patients, parents and physicians [9,31], potentially resulting in inconsistent findings. Therefore ADHD-related difficulties (and thus the impairment) as perceived from various perspectives were assessed in two studies undertaken in Germany in children and adolescents with ADHD [25,32]. The aim of these two studies was to compare the various perspectives as reflected by the newly devised Global Impression of Perceived Difficulties (GIPD) scale. The GIPD can be taken to reflect the difficulties related to ADHD and common co-morbid disorders such as oppositional-defiant disorder (ODD) or conduct disorder (CD) if present. The difficulties captured by the GIPD obviously relate to the degree of impairment, the level of psychosocial functioning and QoL in such children and adolescents at various times of the day [25,32]. Three versions of this scale were used to assess ADHD-related difficulties as perceived from three different perspectives: the patient, parent, and physician perspective. The results of this comparison have been published elsewhere [25,32]. The primary aim of this secondary analysis was to assess the psychometric properties of the GIPD scale in terms of validity and reliability [33]. Using valid and reliable scales is important when measuring QoL in pediatric patients [34-37], especially when assessing children and adolescents with ADHD [38-41].

## Methods

### Study design and procedures

This is a secondary analysis of data from two almost identical multi-center, single-arm, open-label studies in two different age groups (children and adolescents) that were designed to investigate the quality of life in patients with ADHD treated with atomoxetine as reflected by the degree of difficulties perceived by patients, parents and physicians [25,32]. Patients were recruited from child and adolescent psychiatric and pediatric practices and outpatient clinics throughout Germany. Patients aged 6–17 years with ADHD as defined in the Diagnostic and Statistical Manual of Mental Disorders, Fourth Edition, Text Revision (DSM-IV-TR) [1] were eligible for the studies. The diagnosis was confirmed using the "Diagnose-Checkliste Hyperkinetische Störungen" (Diagnostic Checklist for Hyperkinetic Disorders), a structured instrument which is routinely used for the diagnostic assessment of ADHD in Germany [42]. The items of this instrument correspond to those of the ADHD-RS. Patients had to have an IQ of ≥ 70 based on the clinical judgment of the investigator. The exclusion criteria included clinically significant abnormal laboratory findings, acute or unstable medical conditions, cardiovascular disorder, history of seizures, pervasive developmental disorder, psychosis, bipolar disorder, suicidal ideation, any medical condition that might increase sympathetic nervous system activity, or the need for psychotropic medication other than study drug. Patients already being treated with atomoxetine were also excluded. The protocol was approved by an ethics committee, and the study was conducted in accordance with the principles of the Declaration of Helsinki.

Following a wash-out period, baseline assessments were carried out with all the instruments used. During the first week of treatment, the patients received atomoxetine at a dose of approximately 0.5 mg/kg body weight (BW) per day. During the following 7 weeks, the recommended target dose was 1.2 mg/kg BW per day, but could be adjusted within a range of 0.5–1.4 mg/kg BW per day, depending on effectiveness and tolerability. Medication was given once a day in the morning. Assessments were carried out weekly during the first two weeks of treatment, and every two weeks thereafter. After the 8 week treatment period, the physicians decided in accordance with the patients and their parents whether the patient was to continue treatment for additional 16 weeks. Those who participated in this extension period continued on the same atomoxetine dose which again could be adjusted within a range of 0.5–1.4 mg/kg BW per day as considered appropriate by the physician. During the extension period, three assessments were carried out, after 12, 16, and 24 weeks after baseline. The following instruments were used: Global Impression of Perceived Difficulties (GIPD), Attention-Deficit/Hyperactivity Disorder Rating Scale (ADHD-RS), Clinical Global Impression-Severity (CGI-S), and Weekly Rating of Evening and Morning Behavior – Revised (WREMB-R). The data from both studies were combined and analyzed together.

**Figure 1:**
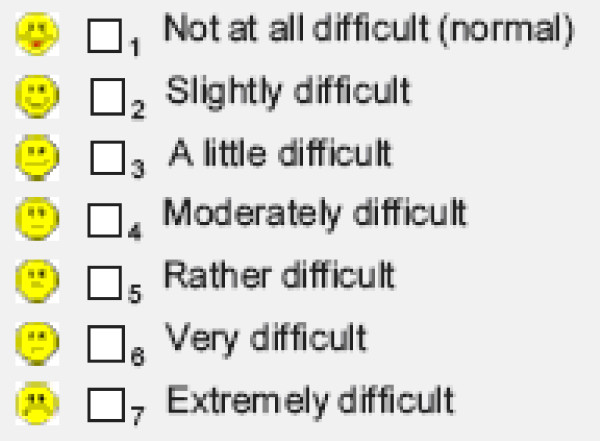
The seven possible answers to each of the five items on the Global Impression of Perceived Difficulties (GIPD) scale as they appear on the report form for each rater (patient, parent, physician).

Table 1 shows the items of the GIPD instrument, which is a five-item rating of ADHD-related difficulties that assesses difficulties in the morning, during school, during homework, in the evening, and overall difficulties over the entire day and night [25]. Each item is rated on a seven point scale (1 = not at all difficult, 7 = extremely difficult) and reflects the situation during the past week (see Figure 1). This instrument was newly devised to detect the perception of the patient's ADHD-related difficulties from a patient, parent (or primary caregiver), and physician perspective. Accordingly, three different versions of the instrument were developed: a patient, a parent, and a physician version, allowing comparison. The GIPD total score was calculated for each rater as the mean of the item scores ranging from 1 to 7. If one item was missing, the total score was also considered to be missing. If the child was unable to fill in the scale on his/her own an independent person (e. g. a study nurse) was allowed to give assistance.

**Table 1:** The five items of the GIPD scale. The wordings of the questions vary slightly, depending on the rater (patient, parent, physician).

**Patient**	**Parent**	**Physician**
1. Think about the past seven days. How difficult have your mornings been?	1. Think about the past seven days. How difficult have the mornings of your child been? Please take into account all information you may have obtained from persons who have also seen your child in the morning.	1. Considering the past seven days, how difficult have the mornings of your patient been? Please include all information provided by the patient and information you may have been able to obtain from other persons who have seen your patient in the morning.
2. Think about the past seven days. How difficult has your time spent in school been?	2. Think about the past seven days. How difficult has the time spent in school been for your child? Please take into account all information you may have obtained from persons who know your child (e.g. parents, teachers, nurses, other caregives).	2. Considering the past seven days, how difficult has the time spent in school been for your patient? Please include all information provided by the patient and information you may have been able to obtain from other persons who know your patient (e.g. parents, teachers, nurses, other caregives).
3. Think about the past seven days. How difficult has your time spent doing homework been?	3. Think about the past seven days. How difficult has the time spent doing homework been for your child? Please take into account all information you may also have obtained from persons who know your child (e.g. parents, teachers, nurses, other caregives).	3. Considering the past seven days, how difficult has the time spent doing homework been for your patient? Please include all information provided by the patient and information you may have been able to obtain from other persons who know your patient (e.g. parents, teachers, nurses, other caregives).
4. Think about the past seven days. How difficult have your evenings been?	4. Think about the past seven days. How difficult have the evenings of your child been? Please take into account all information you may have obtained from persons who have also seen your child in the evening.	4. Considering the past seven days, how difficult have the evenings of your patient been? Please include all information provided by the patient and information you may have been able to obtain from persons who have seen your patient in the evening.
5. Think about the past seven days. How difficult have your days and nights been generally? Did anyone help you with the answers? (yes/no)	5. Think about the past seven days. How difficult have the days and nights of your child been generally? Please take into account all information you may have obtained from other persons who also know your patient (e.g. parents, teachers, nurses, other caregives).	5. Considering the past seven days, how difficult have the days and nights of your patient been generally? Please include all information provided by the patient and information you may have been able to obtain from other persons who know your patient (e.g. parents, teachers, nurses, other caregives).

GIPD = Global Impression of Perceived Difficulties.

The Attention-Deficit/Hyperactivity Disorder Rating Scale-IV-Parent Version: Investigator-Administered and Scored (ADHD-RS) is an 18-item scale, with one item for each of the 18 ADHD symptoms listed in DSM-IV-TR [26,27]. There are two subscales: the "hyperactivity/impulsivity" subscale is the sum of the even items, and the "inattention" subscale is the sum of the odd items. This scale is scored by an investigator while interviewing the parent or primary caregiver. Reliability and validity of this scale has been demonstrated in several European samples including one from Germany [33]

The Clinical Global Impression-Severity-Attention-Deficit/Hyperactivity Disorder scale (CGI-S ADHD) is a seven point single-item rating scale of the clinician's assessment of the severity of ADHD symptoms [28,29].

The WREMB-R-Inv scale is based on the Daily Parent Rating of Evening and Morning Behavior – Revised (DPREMB-R) scale [14]. It has been modified to allow a weekly assessment of behavioral symptoms. In this study, the investigator-rated version was used. The investigator rating was based on information provided by the parent. The DPREMB-R measures 11 specific morning or evening activities (e.g., getting up and out of bed, doing or completing homework, sitting through dinner). The possible score for each item ranges from 0 (no difficulty) to 3 (a lot of difficulty). The DPREMB-R has been validated for the assessment of ADHD behaviors [43] and has been used in several studies to assess behavior in children and adolescents with ADHD [14,15].

### Sample size and statistical analysis

Details on the sample size calculation for the two studies first using the GIPD have been published elsewhere [25,32]. The data of all patients were evaluated (Full Analysis Set, FAS) using SAS version 8. The dataset for all analyses of changes from baseline to endpoint consisted of all patients with a baseline measurement and at least one post-baseline measurement during the 8 week treatment phase.

Evaluation was largely descriptive. All tests of statistical significance were carried out at a nominal level of 5% using two-tailed test procedures. Two-sided confidence intervals (CIs) were computed using a 95% confidence level. All inferences regarding statistical significance were based on comparisons of the 95% confidence intervals (CI). This is equivalent to significance tests with p-values and a two-sided α-level of 5%. To avoid correlations of imputed values, only observed cases (OC) analyses were performed. No imputation of missing values like last observation carried forward (LOCF) was applied.

Percentages of missing values of the GIPD items were calculated for each visit and each perspective. Ceiling and floor effects for the GIPD total score were calculated by the percentage of ratings with the lowest and highest achievable scores for each visit and each perspective. Internal consistency of the GIPD total score was analyzed by using Cronbach's alpha for each visit and each perspective. Additionally, part-whole corrected item-total correlations were provided. Test-retest reliability of the GIPD total score was checked by comparing weeks 6 and 8 in terms of Spearman's correlation coefficient for the items and Pearson's correlation coefficient for the total score for each perspective. The rank-based Spearman's correlation coefficient was used for the items as they have an ordinal structure with only five categories. Pearson's correlation coefficient, which is based on the original values, was used for the total scores in order to assess the linear association of the more continuous total scores. Weeks 6 and 8 were chosen because the treatment and the disease severity was expected to be fairly stable during this period. 95% confidence intervals for the correlation coefficients were computed based on Fisher's z-transformation. Additionally, a weighted version of Cohen's kappa was provided together with 95% CIs.

The validity of the GIPD total score was evaluated as follows: 1) Means over time were provided together with 95% CIs for each perspective. 2) The agreement between the perspectives was described using Cohen's kappa for each visit and each pair of perspectives. 3) The GIPD total score was compared with the WREMB-R total score, the CGI-S score, and the ADHD-RS total score by Pearson's correlation coefficients with 95% CIs for each perspective, at each time point, and for all time points pooled. 4) The GIPD items for morning and evening were compared with the respective sub-scores of the WREMB-R in the same way. 5) Mean GIPD total scores were calculated for each level of the CGI-S with all visits pooled for each perspective to evaluate the relationship between the severity of the disease and the GIPD total score.

## Results

### Patient population and disposition

**Figure 2:**
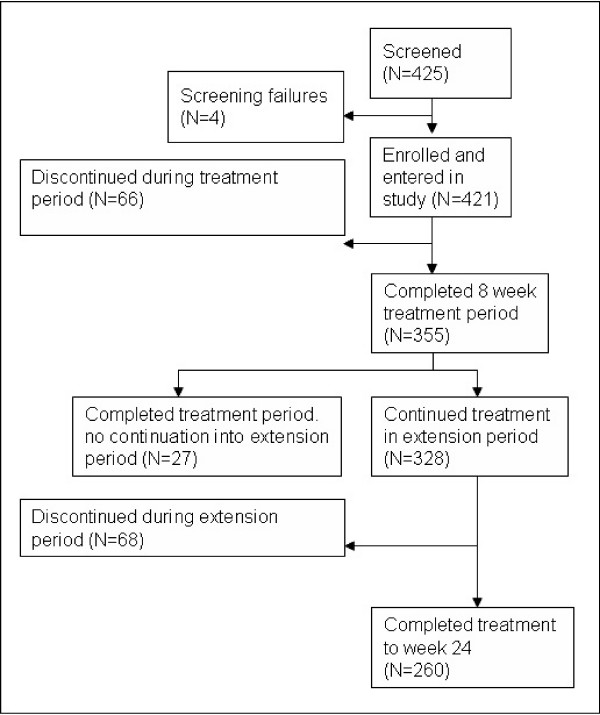
Patient disposition.

Of the 425 patients screened, 421 patients (100%) were enrolled in the two studies and treated with atomoxetine [25,32]. The four patients identified as screening failures initially seemed to be eligible for the study by the investigator. During the baseline visit, it was discovered that the patients did not meet all inclusion criteria or met at least one of the exclusion criteria. The 8-week treatment period was completed by 355 (84.3%) patients. 27 (6.4%) of these did not continue into the extension period because of physician decision. 68 (16.2%) patients discontinued the study between week 8 and week 24. The extension period was completed at week 24 by 260 (61.8%) patients. The reasons for discontinuation were lack of efficacy (12.4%), parent decision (6.9%), adverse event (4.8%), protocol violation (3.6%), patient decision (2.4%), entry criteria exclusion (0.7%), physician decision (0.7%), and patient lost to follow-up (0.5%). The patient disposition is shown in Figure 2.

Table 2 shows the patient characteristics. Boys and patients with combined subtype tended to be younger and were diagnosed earlier than girls or patients with predominantly inattentive subtype. 239 (70.7%) of the boys and 39 (47.0%) of the girls were diagnosed with the combined subtype. The predominantly inattentive subtype was diagnosed in 86 (25.4%) of the boys and 38 (45.8%) of the girls. The subgroups "predominantly hyperactive-impulsive subtype" and "ADHD, not otherwise specified" were too small for subgroup analysis (6 and 13 individuals, respectively).

**Table 2:** Patient characteristics

		**Age (Years)**		**Age at 1st occurrence of symptoms (Years)**		**Age at 1^st ^ADHD-diagnosis (Years)**	
	**N (%)**	**Mean**	**SD**	**Mean**	**SD**	**Mean**	**SD**

All patients	421 (100)	11.1	2.74	4.0	2.03	8.1	2.59
Boys	338 (80.3)	11.0	2.70	4.0	1.94	7.9	2.54
Girls	83 (19.7)	11.6	2.87	4.3	2.35	8.8	2.70
Combined subtype*	278 (66.0)	10.6	2.58	3.7	1.92	7.6	2.42
Predominantly inattentive subtype*	124 (29.5)	12.4	2.59	4.7	1.93	9.2	2.58
Predominantly hyperactive-impulsive subtype*	6 (1.4)	8.6	2.33	4.2	2.01	6.6	2.22
ADHD, not otherwise specified *	13 (3.1)	11.7	3.08	4.8	3.42	9.6	2.30

* According to the Diagnostic and Statistical Manual of Mental Disorders, Fourth Edition

ADHD = Attention-Deficit/Hyperactivity Disorder; SD = standard deviation.

349 (82.9%) of the 421 patients had previously been treated for ADHD. The percentage was similar for the predominantly inattentive subtype (N = 101, 81.5%) and the combined subtype (N = 231, 83.1%). Medications most frequently used were short-acting methylphenidate (N = 290, 68.9%), long-acting methylphenidate (N = 196, 46.6%), amphetamines (N = 56, 13.3%), antipsychotic drugs (N = 12, 2.9%) and herbal/complementary therapies (N = 10, 2.4%). Commonly reported non-drug therapies prior to study were: occupational therapy (N = 48, 11.4%), "other" psychotherapy (N = 31, 7.4%), structured psychotherapy (N = 42, 10.0%), and remedial education (N = 10, 2.4%). The most frequent reason for discontinuation of previous therapy in patients with pre-treatment was inadequate response (N = 216, 61.9%).

The mean dose of atomoxetine given during the first week of treatment was 0.50 mg/kg BW per day (SD 0.07, range 0.40 – 0.80 mg/kg per day). Thereafter, the mean dose for the respective visit intervals ranged between 1.17 and 1.18 mg/kg BW per day (range 0.40 – 1.50 mg/kg day).

Concomitant medication, other concomitant treatments and the presence of any comorbidities in the patient sample are reported elsewhere [25,32].

### Missing values

For patients, missing values were between 0 and 0.38% for items 1, 4, and 5, and between 0.24% and 1.61% for items 2 and 3. For parents, missing values for the morning and evening ratings (items 1 and 4) were between 0 and 0.25% of all ratings for the respective items and the various visits. For items 2, 3, and 5 missing values occurred between 0 and 3.13% of all ratings for the respective item and visit. For physicians, there were no missing values for items 1, 4, and 5 at all but one visit. For items 2 and 3 there were missing values between 0 and 2.1% of all ratings for the respective item and visit. Thus, the items related to school and homework were those with the highest percentage of missing values. However, the percentage of missing values did not exceed 3.1%, indicating a tolerable lack of information. For the other three items, missing values were negligible.

### Floor and ceiling effects

At baseline, floor effects (GIPD total score = 1) were 15.38% for the patient, 2.91% for the parent, and 1.45% for the physician perspective. The ceiling effects (GIPD total score = 7) were 0.96% for the patient, 2.18% for the parent, and 0.97% for the physician perspective. At endpoint (week 24) floor effects increased to 45.17% for the patient, 26.07% for the parent, and 27.59% for the physician perspective (as would be expected after successful treatment). The ceiling effects decreased to 0.39% for the patient, 0.39% for the parent, and 0% for the physician perspective.

### Internal consistency

Table 3 shows the internal consistencies (Cronbach's alpha) of the GIPD total scores reflecting the ratings of the patients, the parents and the physicians at baseline and the following 8 points in time. Except for the first rating by the patients, all alpha values were above 0.80, indicating a good to excellent internal consistency of the scale. None of the consistency scores could be increased by deleting one of the items. The part-whole corrected item-total correlations were found to be above 0.46 for all weeks and raters, indicating moderate to good item-total Pearson's correlations of all items at all assessment points.

**Table 3:** Internal consistency (Cronbach's alpha) and part-whole corrected item-total Pearson's correlation coefficient (minimum and maximum of the 5 items) in brackets for the GIPD-Total Score (OC) over time

**Week**	**Patient**	**Parent**	**Physician**
0	0.76 (0.46 – 0.67)	0.84 (0.57 – 0.80)	0.86 (0.57 – 0.86)
1	0.83 (0.55 – 0.72)	0.87 (0.62 – 0.86)	0.90 (0.69 – 0.89)
2	0.82 (0.52 – 0.72)	0.90 (0.68 – 0.88)	0.91 (0.67 – 0.92)
4	0.83 (0.53 – 0.74)	0.89 (0.67 – 0.88)	0.90 (0.67 – 0.90)
6	0.85 (0.56 – 0.78)	0.90 (0.67 – 0.89)	0.91 (0.69 – 0.91)
8	0.83 (0.50 – 0.78)	0.88 (0.64 – 0.85)	0.90 (0.65 – 0.91)
12	0.82 (0.52 – 0.71)	0.91 (0.70 – 0.89)	0.93 (0.75 – 0.93)
16	0.82 (0.52 – 0.78)	0.90 (0.69 – 0.88)	0.92 (0.72 – 0.91)
24	0.83 (0.57 – 0.76)	0.89 (0.66 – 0.87)	0.91 (0.66 – 0.92)

GIPD = Global Impression of Perceived Difficulties; OC = observed cases.

### Test-retest reliability

Spearman's correlations between items rated at weeks 6 and 8 ranged from 0.473 [CI 0.380 to 0.554] (item 1) to 0.557 [CI 0.474 to 0.629] (item 5) for the patient, 0.551 [CI 0.466 to 0.624] (item 2) and 0.600 [CI 0.521 to 0.667] (item 4) for the parent, 0.525 [CI 0.439 to 0.600] (item 2) and 0.578 [CI 0.498 to 0.647] (item 5) for the physician perspective. The respective Cohen's kappas were between 0.410 [CI 0.278 to 0.542] (item 3) and 0.597 [CI 0.505 to 0.688] (item 5) for the patient, 0.509 [CI 0.410 to 0.609] (item 2) and 0.607 [CI 0.511 to 0.704] (item 1) for the parent, and between 0.543 [CI 0.433 to 0.652] (item 3) and 0.580 [CI 0.487 to 0.673] (item 5) for the physician perspective.

Pearson's correlation coefficients of the total scores rated at weeks 6 and 8 were 0.644 [CI 0.573 to 0.704] for the patient, 0.670 [CI 0.602 to 0.728] for the parent, and 0.654 [CI 0.584 to 0.713] for the physician perspective. The respective Cohen's kappas were 0.642 [CI 0.555 to 0.729], 0.669 [CI 0.589 to 0.749], and 0.653 [CI 0.559 to 0.746]. These results indicate a moderate to good test-retest reliability within a period of two weeks.

## Validity

### Agreement between patient, parent and physician perspectives over time (convergent validity)

**Figure 3:**
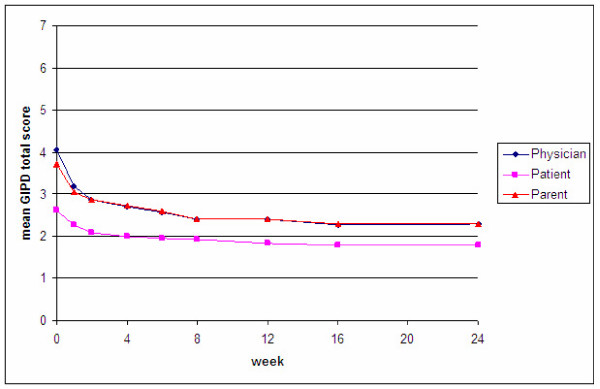
Mean GIPD total score (OC) over time for the three perspectives (patient, parent, physician).

The courses of the mean GIPD total scores over time (OC) were generally parallel in terms of the three rater groups for all patients (Figure 3).

Parents rated the ADHD-related difficulties at baseline as significantly less severe than physicians (mean GIPD total score, baseline parent: 18.6 [CI 18.0 to 19.2]), physician: 20.3 [CI 19.7 to 20.8]). However, the parent and physician total scores converged as early as week 2 (parent: 14.3 [CI 13.6 to 14.9], physician: 14.3 [CI 13.7 to 14.9]) and overlapped for the remainder of the observation period (week 24, parent: 11.5 [CI 10.8 to 12.2], physician: 11.5 [CI 10.9 to 12.2]). Compared to the parent and physician ratings, the children and adolescents perceived their difficulties as significantly less severe over the entire observation period (mean GIPD-total score patient baseline: 13.1 [CI 12.5 to 13.6], week 2: 10.4 [CI 9.9 to 11.0], week 24: 8.9 [CI 8.3 to 9.4]).

At baseline, Cohen's kappa for the GIPD total scores revealed the highest agreement between physician and parent ratings (kappa baseline: 0.521 [CI: 0.471 to 0.572]). Over all, the degree of agreement increased over time (kappa week 8: 0.653 [CI: 0.606 to 0.700], kappa week 24: 0.661 [CI: 0.609 to 0.712]). Agreement between patients and parents as well as patients and physicians was almost identical at baseline (kappa patient/physician baseline: 0.142 [CI: 0.106 to 0.178]; kappa patient/parent baseline: 0.162 [CI: 0.116 to 0.207]). Agreement between patient ratings and parent or physician ratings increased from baseline to week 8 and to week 24 (kappa patient/physician week 8: 0.327 [CI: 0.258 to 0.395]; kappa patient/parent week 8: 0.312 [CI: 0.239 to 0.386], kappa patient/physician week 24: 0.319 [CI: 0.253 to 0.385]; kappa patient/parent week 24: 0.262 [CI: 0.196 to 0.328]). Agreement between patients and physicians as well as between patients and parents was significantly lower than agreement between physicians and parents at any point in time (Table 4).

**Table 4:** Cohen's kappa for GIPD total scores (OC) with 95% confidence intervals (CI) over time.

**Week**	**Patient – Parent**	**Patient – Physician**	**Physician – Parent**
0	0.1615 (0.116;0.207)	0.1420 (0.106;0.178)	0.5212 (0.471;0.572)
1	0.1992 (0.144;0.254)	0.1968 (0.150;0.244)	0.5748 (0.522;0.627)
2	0.2464 (0.189;0.304)	0.2605 (0.206;0.315)	0.6282 (0.581;0.676)
4	0.2318 (0.176;0.288)	0.2790 (0.221;0.337)	0.6198 (0.572;0.667)
6	0.2493 (0.185;0.313)	0.3036 (0.244;0.363)	0.6777 (0.636;0.719)
8	0.3124 (0.239;0.386)	0.3269 (0.258;0.395)	0.6530 (0.606;0.700)
12	0.2287 (0.172;0.286)	0.2361 (0.181;0.291)	0.6373 (0.586;0.689)
16	0.2569 (0.187;0.327)	0.3150 (0.245;0.385)	0.6348 (0.579;0.690)
24	0.2618 (0.196;0.328)	0.3188 (0.253;0.385)	0.6607 (0.609;0.712)

GIPD = Global Impression of Perceived Difficulties; OC = observed cases.

### Comparison of GIPD total scores with WREMB-R (convergent validity), CGI-S, and ADHD-RS total scores (discriminant validity) over time

As shown in Table 5, the correlation between GIPD total score and WREMB-R total score from the three perspectives at baseline was significantly lower from a patient perspective (0.265, 95% CI: 0.173 to 0.352) than from a parent (0.554, 95% CI: 0.482 to 0.617) or physician perspective (0.675, 95% CI: 0.618 to 0.724). Over time, this pattern persisted, although with slightly higher correlations. At week 24, correlation of the GIPD with the WREMB-R was significantly lower from a patient perspective (0.381, 95% CI: 0.271 to 0.480) than from a parent (0.731, 95% CI: 0.667 to 0.783) or physician perspective (0.774, 95% CI: 0.720 to 0.818).

**Table 5:** Comparison of GIPD total scores with WREMB-R, CGI-S, and ADHD-RS total scores over time (Pearson's Correlation Coefficients with 95% CIs).

	**Week**	**GIPD total score (patient-rated)**	**GIPD total score (parent-rated)**	**GIPD total score (physician-rated)**
WREMB-R total score	0	0.265 (0.173;0.352)	0.554 (0.482;0.617)	0.675 (0.618;0.724)
	1	0.299 (0.207;0.384)	0.660 (0.600;0.712)	0.728 (0.678;0.770)
	2	0.345 (0.255;0.428)	0.717 (0.665;0.762)	0.772 (0.729;0.808)
	4	0.422 (0.335;0.500)	0.710 (0.656;0.756)	0.764 (0.719;0.803)
	6	0.369 (0.276;0.454)	0.745 (0.695;0.787)	0.784 (0.740;0.820)
	8	0.421 (0.327;0.506)	0.733 (0.677;0.779)	0.779 (0.731;0.818)
	12	0.340 (0.238;0.434)	0.765 (0.714;0.807)	0.789 (0.743;0.827)
	16	0.418 (0.313;0.511)	0.708 (0.642;0.763)	0.793 (0.744;0.833)
	24	0.381 (0.271;0.480)	0.731 (0.667;0.783)	0.774 (0.720;0.818)
	pooled	0.409 (0.380;0.438)	0.736 (0.719;0.752)	0.799 (0.786;0.811)

CGI-S	0	0.276 (0.185;0.363)	0.407 (0.323;0.485)	0.570 (0.501;0.631)
	1	0.291 (0.200;0.377)	0.508 (0.431;0.577)	0.651 (0.591;0.703)
	2	0.305 (0.213;0.391)	0.524 (0.447;0.592)	0.671 (0.613;0.721)
	4	0.374 (0.284;0.457)	0.575 (0.504;0.638)	0.693 (0.636;0.741)
	6	0.399 (0.309;0.482)	0.655 (0.591;0.710)	0.741 (0.691;0.784)
	8	0.380 (0.283;0.469)	0.579 (0.501;0.646)	0.723 (0.666;0.770)
	12	0.297 (0.193;0.395)	0.621 (0.547;0.684)	0.699 (0.637;0.750)
	16	0.363 (0.254;0.461)	0.571 (0.483;0.646)	0.723 (0.660;0.774)
	24	0.312 (0.197;0.418)	0.578 (0.490;0.654)	0.674 (0.600;0.734)
	pooled	0.391 (0.361;0.420)	0.616 (0.594;0.637)	0.733 (0.717;0.749)

ADHD-RS total score	0	0.201 (0.106;0.291)	0.427 (0.344;0.503)	0.514 (0.439;0.581)
	1	0.236 (0.142;0.325)	0.573 (0.503;0.635)	0.670 (0.612;0.720)
	2	0.309 (0.217;0.395)	0.638 (0.575;0.693)	0.692 (0.637;0.740)
	4	0.385 (0.296;0.467)	0.659 (0.597;0.712)	0.708 (0.654;0.754)
	6	0.322 (0.226;0.410)	0.650 (0.585;0.705)	0.676 (0.616;0.728)
	8	0.323 (0.222;0.417)	0.645 (0.576;0.704)	0.693 (0.631;0.745)
	12	0.251 (0.144;0.352)	0.653 (0.583;0.712)	0.648 (0.579;0.707)
	16	0.334 (0.223;0.435)	0.689 (0.619;0.746)	0.737 (0.677;0.787)
	24	0.311 (0.196;0.417)	0.661 (0.585;0.724)	0.680 (0.608;0.740)
	pooled	0.356 (0.325;0.387)	0.666 (0.646;0.685)	0.723 (0.705;0.739)

GIPD = Global Impression of Perceived Difficulties; WREMB-R = Weekly Rating of Evening and Morning Behavior – Revised; CGI-S = Clinical Global Impression – Severity; ADHD-RS = Attention-Deficit/Hyperactivity Disorder Rating Scale; 95% CI = 95% Confidence Interval.

Correlation between the physician-rated CGI-S total score and GIPD total score from the three perspectives at baseline was significantly lower both from a patient (0.269, 95% CI: 0.185 to 0.363) and a parent perspective (0.407, 95% CI: 0.323 to 0.485) than from a physician perspective (0.570, 95% CI: 0.501 to 0.631). Over time, this pattern persisted, although with slightly higher correlations. However, the differences between parent and physician ratings were not always statistically significant. At endpoint, correlation was significantly lower from a patient perspective (0.312, 95% CI: 0.197 to 0.418) than from a physician perspective (0.674, 95% CI: 0.600 to 0.734). The correlation was 0.578 (95% CI: 0.490 to 0.654) from a parent perspective and therefore significantly higher than from a patient perspective.

Comparing the confidence intervals (CI), the correlation between the physician-rated ADHD-RS total score and GIPD total score from the three perspectives at baseline was significantly lower from a patient perspective (0.201, 95% CI: 0.106 to 0.291) than from a parent (0.427, 95% CI: 0.344 to 0.503) or physician perspective (0.514, 95% CI: 0.439 to 0.581). Over time, this pattern persisted, although with slightly higher correlations. At week 24, correlation was significantly lower from a patient perspective (0.311, 95% CI: 0.196 to 0.417) than from a parent (0.661, 95% CI: 0.585 to 0.724) or physician perspective (0.680, 95% CI: 0.608 to 0.740).

### Comparison of GIPD scores for the morning and evening items with the WREMB-R morning and evening subscales (convergent validity)

As shown in Table 6, the comparison of GIPD scores for the morning and evening items with the WREMB-R morning and evening subscales showed a similar pattern as was seen with the GIPD and WREMB-R total scores.

**Table 6:** Comparison of GIPD scores for the morning and evening items with the WREMB-R morning and evening subscales (Pearson's Correlation Coefficients with 95% CIs).

	**Week**	**GIPD morning (patient-rated)**	**GIPD morning (parent-rated)**	**GIPD morning (physician-rated)**
WREMB-R morning subscore	0	0.258 (0.166;0.346)	0.612 (0.547;0.668)	0.714 (0.663;0.758)
	1	0.442 (0.361;0.517)	0.644 (0.582;0.698)	0.727 (0.677;0.769)
	2	0.318 (0.226;0.403)	0.699 (0.644;0.746)	0.760 (0.714;0.798)
	4	0.311 (0.217;0.398)	0.754 (0.706;0.794)	0.742 (0.693;0.783)
	6	0.297 (0.200;0.388)	0.738 (0.686;0.781)	0.778 (0.734;0.815)
	8	0.331 (0.231;0.424)	0.677 (0.612;0.731)	0.776 (0.729;0.816)
	12	0.332 (0.229;0.426)	0.734 (0.678;0.781)	0.791 (0.746;0.829)
	16	0.361 (0.252;0.460)	0.710 (0.645;0.764)	0.779 (0.727;0.821)
	24	0.313 (0.198;0.418)	0.734 (0.671;0.785)	0.778 (0.725;0.822)
	pooled	0.371 (0.341;0.401)	0.719 (0.701;0.735)	0.786 (0.772;0.799)

		**GIPD evening (patient-rated)**	**GIPD evening (parent-rated)**	**GIPD evening (physician-rated)**

WREMB-R evening subscore	0	0.321 (0.231;0.404)	0.537 (0.464;0.602)	0.695 (0.641;0.741)
	1	0.276 (0.184;0.363)	0.625 (0.561;0.681)	0.717 (0.665;0.760)
	2	0.337 (0.247;0.421)	0.686 (0.630;0.735)	0.790 (0.750;0.824)
	4	0.348 (0.257;0.433)	0.672 (0.612;0.723)	0.767 (0.722;0.805)
	6	0.398 (0.307;0.480)	0.774 (0.728;0.812)	0.790 (0.748;0.826)
	8	0.270 (0.166;0.368)	0.731 (0.675;0.778)	0.772 (0.723;0.812)
	12	0.177 (0.067;0.282)	0.684 (0.619;0.738)	0.707 (0.647;0.758)
	16	0.347 (0.237;0.447)	0.683 (0.612;0.741)	0.764 (0.709;0.809)
	24	0.336 (0.223;0.440)	0.733 (0.670;0.784)	0.767 (0.711;0.812)
	pooled	0.351 (0.320;0.382)	0.708 (0.690;0.725)	0.790 (0.776;0.803)

GIPD = Global Impression of Perceived Difficulties; WREMB-R = Weekly Rating of Evening and Morning Behavior – Revised; 95% CI = 95% Confidence Interval.

### Mean GIPD total scores by symptom severity (CGI-S) (discriminant validity)

**Figure 4:**
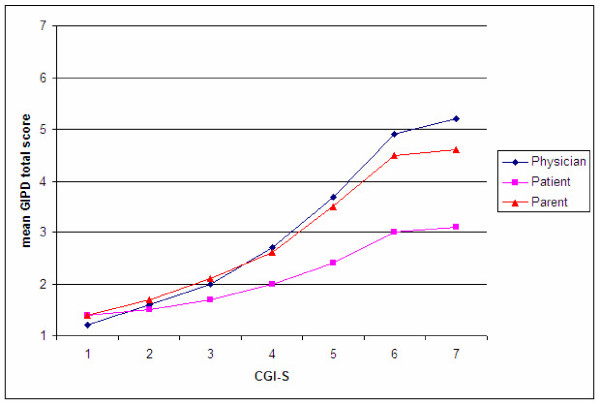
Mean GIPD total scores (OC) by CGI-S levels (OC) based on all time points for the three perspectives (patient, parent, physician).

When relating GIPD total scores to the seven severity levels on the CGI-S, a monotone but not linear increase can be found. Both parent and physician GIPD total scores increased similarly with increasing CGI-S scores (Figure 4). In contrast, the patient-rated mean GIPD total scores increased to a much lower degree with increasing CGI-S scores.

## Discussion

The aim of this post-hoc analysis was to evaluate and validate the newly devised GIPD scale that measures the degree of ADHD-related difficulties perceived by patients, parents and physicians at various times of the day. Other scales available so far to assess the level of psychosocial functioning from several perspectives, such as the Child Health Questionnaire (CHQ) [44], Child Health and Illness Profile (CHIP) [45], or the Marburg System of Quality Assurance and Therapy Evaluation (MARSYS) [46,47], do not distinguish between various times of the day. Furthermore, the GIPD can be taken to reflect the health-related quality of life in children and adolescents with ADHD [25]. However, since the introduction of various long-acting ADHD medications, interest in duration of action of these medications over the day has increased [12]. The GIPD was therefore designed both to capture ADHD-related difficulties and to distinguish between various times of the day. Thus, results obtained by using the GIPD can be compared with results obtained by using other instruments that distinguish between various times of the day, such as the Daily Parent Rating of Evening and Morning Behavior (DPREMB) scale [14] or the Weekly Rating of Evening and Morning Behavior (WREMB) scale [43]. As the GIPD was designed as a global impression scale, all five items of the GIPD resemble the Clinical Global Impression (CGI) scale [28,29] quite closely.

A total of 421 children and adolescents diagnosed with ADHD according to DSM-IV criteria were included in this analysis of data from two open-label studies. The mean age of the patients was 11.1 (SD 2.74) years, 338 (80.3%) were boys, 83 (19.7%) were girls.

One of the findings of the two studies on which the GIPD validation is based was a relatively high percentage of patients with the predominantly inattentive type of ADHD. One can only speculate about the reasons. Perhaps there was some sort of selection bias in these open-label studies towards patients with the inattentive type, particularly the study with adolescents. In the study with children, only 19.5% of patients were of the inattentive type, whilst considerable 45.9% were of the inattentive type in the study with adolescents. This obviously resulted in a proportion of 29.5% with the inattentive type in the pooled data (children and adolescents). One further reason could be the fact that the symptoms change as patients grow older: hyperactive or impulsive behaviour tends to decline, whilst inattentive symptoms tend to remain. This would explain the greater proportion of patients with the inattentive subtype in the study with adolescents compared to the study with children.

Although it is short (5 items), the GIPD scale rated by parents, physicians and patients has been shown to be internally consistent. For all items at least a moderate item-total correlation was found. Moreover, the scale has also shown good test-retest reliability over a period of two weeks for all three perspectives. The mean GIPD total scores (OC) generally showed a parallel course over time from all three rater perspectives (Figure 3). Parents rated ADHD-related difficulties at baseline as significantly less severe than physicians, but the parent and physician total scores converged as early as week 2 and the Cohen's kappa coefficients indicate a moderate agreement between parents' and physicians' ratings. However, the agreement between parents and physicians on the one hand and the patients on the other hand were quite low, as indicated by the kappa coefficients. Thus, adults seem to agree to a greater extent on the degree of perceived ADHD-related difficulties in the patients than the children and adolescents.

However, patients, parents and physicians perceived an improvement of ADHD-related difficulties over time. Compared to the parent and physician ratings, the children and adolescents perceived their difficulties as significantly less severe throughout the entire study. This suggests that children and adolescents perceive their ADHD-related difficulties to a lower extent than adults do. These findings reflect the findings from the two studies that assessed children and adolescents separately [25,32]. Moreover, studies on the correlations between the ratings of behavioral and emotional problems as rated by parents and children or adolescents also reveal little agreement in the ratings of parents and their children. For example, Achenbach et al. [48] found in their meta-analysis a correlation of r = 0.25 between parents and children ratings of behavioral and emotional problems. This result was replicated in a German sample [49].

The higher correlation between parent and physician perspectives may also be due to the fact that the physicians based their ratings primarily on the information from the parents rather than the patients. Thus, convergent validity may be artificially inflated. However, the patient perspective on daily difficulties provides important additional information when evaluating the efficacy and effectiveness of a treatment. The low to moderate correlations of the different perspectives underline the need for assessing these perspectives separately.

The moderate correlations between physician-rated ADHD symptoms on the ADHD rating scale and the GIPD indicate a reasonable discriminant validity of the difficulties and the impairment of the child in different settings throughout the day as assessed by the GIPD scale on the one hand and the ADHD-RS on the other. Somewhat higher correlations were found with the WREMB-R which assesses a similar construct (i. e. 11 specific common morning or evening behaviors). This finding indicates the convergent validity of the GIPD in showing higher correlations to scales assessing similar constructs.

These studies and analyses have several limitations. Most importantly, they did not include a placebo control, so that the degree to which the results reflect drug-specific effects cannot be determined definitively. Also, sensitivity regarding differences between placebo and active comparator cannot be determined. In these studies, no further instrument assessing behavioral or emotional problems as perceived by the patients were used. Such self-report scales on ADHD symptoms or ADHD-related difficulties allow the calculation of convergent and discriminant validity and allow comparisons with other self report measures [42]. A future comparison of this sample with children without ADHD or with other behavioral or emotional problems would be interesting. This would allow the assessment of perceived difficulties in a more representative sample. A further limitation of this study is the age-distribution of the sample that does not reflect the age-distribution of individuals with ADHD in the general population. This is due to the fact that this analysis is based on two identical studies, one in children and one in adolescents. Beyond age as a covariate, other factors such as ADHD subtype, co-morbid disorders, type of school, family environment or other environmental factors may also influence a range of GIPD results (e.g. agreement between perspectives or treatment response as reflected by the GIPD). Further research on these factors is warranted.

Treatment-emergent adverse events during the course of the two studies have been reported elsewhere [25,32].

Overall, the GIPD can be considered an internally consistent, reliable and valid measure to assess difficulties experienced by children with ADHD throughout the day and can be used as an indicator for psychosocial impairment and quality of life [25]. Moreover, the two treatment studies on which this analysis is based also show that the scale is sensitive to treatment-related change.

## Competing interests

Research was funded by Lilly Deutschland GmbH, Bad Homburg, Germany. Dr. Peter M. Wehmeier (PMW), Prof. Ralf W. Dittmann (RWD) and Dr. Alexander Schacht (AS) are full-time employees of Lilly Deutschland. Prof. Manfred Döpfner (MD) has received research grants and speaker honoraria from Eli Lilly and he is a member of several Lilly Advisory Boards.

## Authors' contributions

PMW, RWD, and AS developed the two clinical trials, AS developed the analyses used for this manuscript. All authors participated in development of the GIPD scale and the interpretation of data, PMW and AS drafted the manuscript, RWD and MD revised it critically for important intellectual content. All authors read and approved the final manuscript.
